# Epidemiology of traumatic cervical spinal fractures in a general Norwegian population

**DOI:** 10.1186/s40621-022-00374-w

**Published:** 2022-03-24

**Authors:** Nils Christian Utheim, Eirik Helseth, Mona Stroem, Paal Rydning, Magnus Mejlænder-Evjensvold, Thomas Glott, Christina Teisner Hoestmaelingen, Mads Aarhus, Paal Andre Roenning, Hege Linnerud

**Affiliations:** 1 Department of Neurosurgery, Oslo University Hospital, Nydalen, P. O. Box 4956, 0424 Oslo, Norway; 2 Institute of Clinical Medicine, Faculty of Medicine, University of Oslo, Oslo, Norway; 3 Spinal Unit, Sunnaas Rehabilitation Hospital, Nesodden, Norway; 4 Department of Neuroradiology, Oslo University Hospital, Oslo, Norway

**Keywords:** Spinal fracture, Spinal cord injury, Cervical, Traumatic, Epidemiology, Population-based

## Abstract

**Background:**

In Western countries, the typical cervical spine fracture (CS-Fx) patient has historically been a young male injured in a road traffic accident. Recent reports and daily clinical practice clearly indicate a change in the typical patient from a young male to an elderly male or female with comorbidities. This study aimed to establish contemporary population-based epidemiological data of traumatic CS-Fx for use in health-care planning and injury prevention.

**Methods:**

This is a population-based retrospective database study (with prospectively collected data) from the Southeast Norway health region with 3.0 million inhabitants. We included all consecutive cases diagnosed with a CS-Fx between 2015 and 2019. Information regarding demographics, preinjury comorbidities, trauma mechanisms, injury description, treatment, and level of hospital admittance is presented.

**Results:**

We registered 2153 consecutive cases with CS-Fx during a 5-year period, with an overall crude incidence of CS-Fx of 14.9/100,000 person-years. Age-adjusted incidences using the standard population for Europe and the World was 15.6/100,000 person-years and 10.4/100,000 person-years, respectively. The median patient age was 62 years, 68% were males, 37% had a preinjury severe systemic disease, 16% were under the influence of ethanol, 53% had multiple trauma, and 12% had concomitant cervical spinal cord injury (incomplete in 85% and complete in 15%). The most common trauma mechanisms were falls (57%), followed by bicycle injuries (12%), and four-wheel motorized vehicle accidents (10%). The most common upper CS-Fx was C2 odontoid Fx, while the most common subaxial Fx was facet joint Fx involving cervical level C6/C7. Treatment was external immobilization with a stiff neck collar alone in 65%, open surgical fixation in 26% (giving a 3.7/100,000 person-years surgery rate), and no stabilization in 9%. The overall 90-day mortality was 153/2153 (7.1%).

**Conclusions:**

This study provides an overview of the extent of the issue and patient complexity necessary for planning the health-care management and injury prevention of CS-Fx. The typical CS-Fx patient was an elderly male or female with significant comorbidities injured in a low-energy trauma. The overall crude incidences of CS-Fx and surgical fixation of CS-Fx in Southeast Norway were 14.9/100,000 person-years and 3.7/100,000 person-years, respectively.

## Background

The reported incidence of traumatic cervical spine fracture (CS-Fx) in general Western populations ranges from 4–17/100,000 person-years (Brolin and Holst 2002; Fredo et al. 2014, 2012; Hackenberg et al. 2022; Hu et al. 1976; Kumar et al. 2018; Niemi-Nikkola et al. 1976; Roche et al. 2008), and the proportion of concomitant cervical spinal cord injury (cSCI) is in the range of 10–11% (Fredo et al. 2014; Hackenberg et al. 2022).

The typical CS-Fx patient has historically been a young male injured in a road traffic accident (Ryan and Henderson 1992). Recent reports and daily clinical practice indicated a change in the typical patient from a young male injured in a high-energy trauma to an elderly male or female with significant comorbidities injured in a low-energy trauma, similar to what has been observed for patients with traumatic brain injury (Fredo et al. 2014, 2012; Niemi-Nikkola et al. 1976; Tverdal et al. 2020; Maas et al. 2017; Steyerberg et al. 2019). These patients’ total care burden depends on several factors: the incidence of CS-Fx, patient age and comorbidities, concomitant cervical spinal cord injury (cSCI), multiple concomitant trauma, mode of fracture stabilization (external immobilization or surgical fixation), and need for specialized rehabilitation. Furthermore, injury prevention efforts must be updated to reflect the typical patient and the most frequent injury mechanisms.

Here, we present a contemporary population-based prospective epidemiological study of traumatic CS-Fx in Southeast Norway with a population of 3.0 million people covering the period of 2015–2019. We will discuss the implications for health-care planning and injury prevention.

## Methods

Oslo University Hospital (OUH) is a level 1 trauma center situated in Oslo and serves as the major trauma care facility in the Southeast Norway health region. OUH is the only hospital in this region with a neurosurgical service. OUH performs > 95% of the trauma-related neurosurgical procedures in this population, including all surgeries for cervical spine injury. In Norway, all surgical procedures for cervical spine injuries are done by neurosurgeons. There are 20 hospitals within the Southeast Norway health region with general and/or orthopedic surgeons and radiological services that refer patients with head and cervical spine injuries to OUH. Either the patients were admitted to OUH for surgical treatment, or nonsurgical treatment was carried out locally after consultation with the Department of Neurosurgery. In 2019, the Southeast Norway health region had 3.0 million inhabitants. A detailed description of the Norwegian population concerning sex, age, and county can be found at www.ssb.no.

This is a retrospective database study (with prospectively collected data) of all consecutive cases included in our quality control database for traumatic CS-Fx in Southeast Norway from January 1, 2015, to December 31, 2019. In the database, we prospectively registered all CS-Fx patients (C0/C1 to C7/Th1) diagnosed with cervical CT (100%) and/or cervical MRI in Southeast Norway. Only patients with an 11-digit unique Norwegian Social Security Number living within Southeast Norway were included. Cervical MRI was performed in 1370/2153 (63.6%) of the patients and in 240/249 (96.4%) patients with cSCI. After diagnosing at CS-Fx at any of the local hospitals in the Southeast Norway health region, the treating physician at the local hospital consult the neurosurgeon on call at the Department of Neurosurgery, OUH and a decision is made either to transfer the patient to OUH or to start conservative treatment locally. The neurosurgeon on call at OUH inform the deputy registrar of the CS-Fx registry, who will do a preliminary registration of the patient. (The role as deputy registrar rotates among the residents/neurosurgeons on a weekly basis.) Once a week, the main registrar receives a list of all new CS-Fx for the last week and enter them into the CS-Fx registry. In order to identify potential missed patients, the main registrar every week reviews the surgical protocol for the last week and all last week outpatient consultations for CS-Fx. The completeness of the registry is 100% for surgically treated CS-Fx, since OUH is the only hospital in Southeast Norway performing surgical fixation of CS-Fx. The registry is incomplete for non-surgically treated CS-Fx due to some undiagnosed fractures and potential underreporting of CS-Fx from local hospitals.

The following data were retrieved from the database: date of injury, sex, age at time of injury, living status at time of injury (home—care for self, home—but need assistance with activities of daily life (ADL), or institutionalized), preinjury ASA score (American Society of Anesthesiologists Physical Status Classification system) (Skaga et al. 2007) (1: normal healthy; 2: mild systemic disease; 3: severe systemic disease; 4: life threatening systemic disease), trauma mechanism, anatomical level of cervical injury dichotomized into C0–C2 and C3–C7 (subaxial), C0-C2 CS-Fxs subclassified by fracture morphology or with type of dislocation in cases with dominating ligamentous injury, subaxial CS-Fxs subclassified by level of “suspected instability” and whether or not the facet joint was injured, cSCI classified according to the ASIA Impairment Scale (AIS) (Roberts et al. 2017) into grade A (complete)—B-C-D (incomplete)—E (none), multiple trauma (no/monotrauma or yes/polytrauma), concomitant head injury was scored according to Head Injury Severity Score (HISS) (Stein and Spettell 1995) into mild-moderate-severe, concomitant thoracolumbar fracture (no or yes), concomitant thoracolumbar SCI (no or yes), treatment of CS-Fx (conservative versus surgical), OUH management role (admitted to OUH, image review and treatment advice from OUH or follow-up at OUH after primary treatment elsewhere), length of primary hospital stay at OUH (LOS), and 90-day mortality.

Multiple trauma was defined as a simultaneous traumatic brain injury (mild, moderate, or severe according to HISS) and/or imaging-proven (X-ray, CT, or ultrasound) injury in one or more of the following regions: face, thoracolumbar spine, chest, abdomen, pelvis or extremities. Skin injuries were not registered.

Data were summarized using frequencies for categorical data and median values for continuous data and if data were not normally distributed or skewed. The Wilcoxon rank-sum test, Kruskal–Wallis test, and Chi-squared tests were used to compare continuous and categorical variables. Uni- and multivariate binary logistic regression analyses were used to investigate the effect of different covariates on 90-day mortality. R v 4.0 was used for all statistical analyses (R Core Team. R 2020). P values < 0.05 were considered significant. Incidence per 100,000 was calculated in person years. For age-adjusted incidence according to the direct method, we used the 2013 European standard population (ESP) and the 2000–2025 WHO World standard population.

## Results

### Incidence of cervical spine fracture (CS-Fx)

**Fig. 1:**
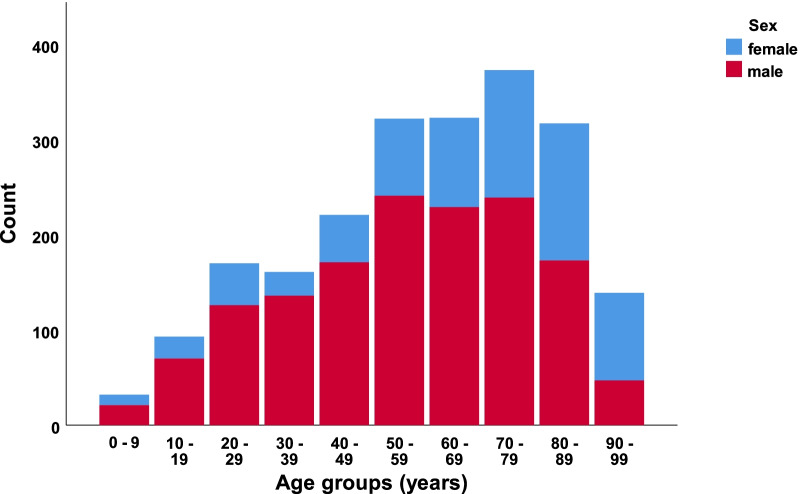
Number of cervical spine fractures according to age group and sex

**Fig. 2:**
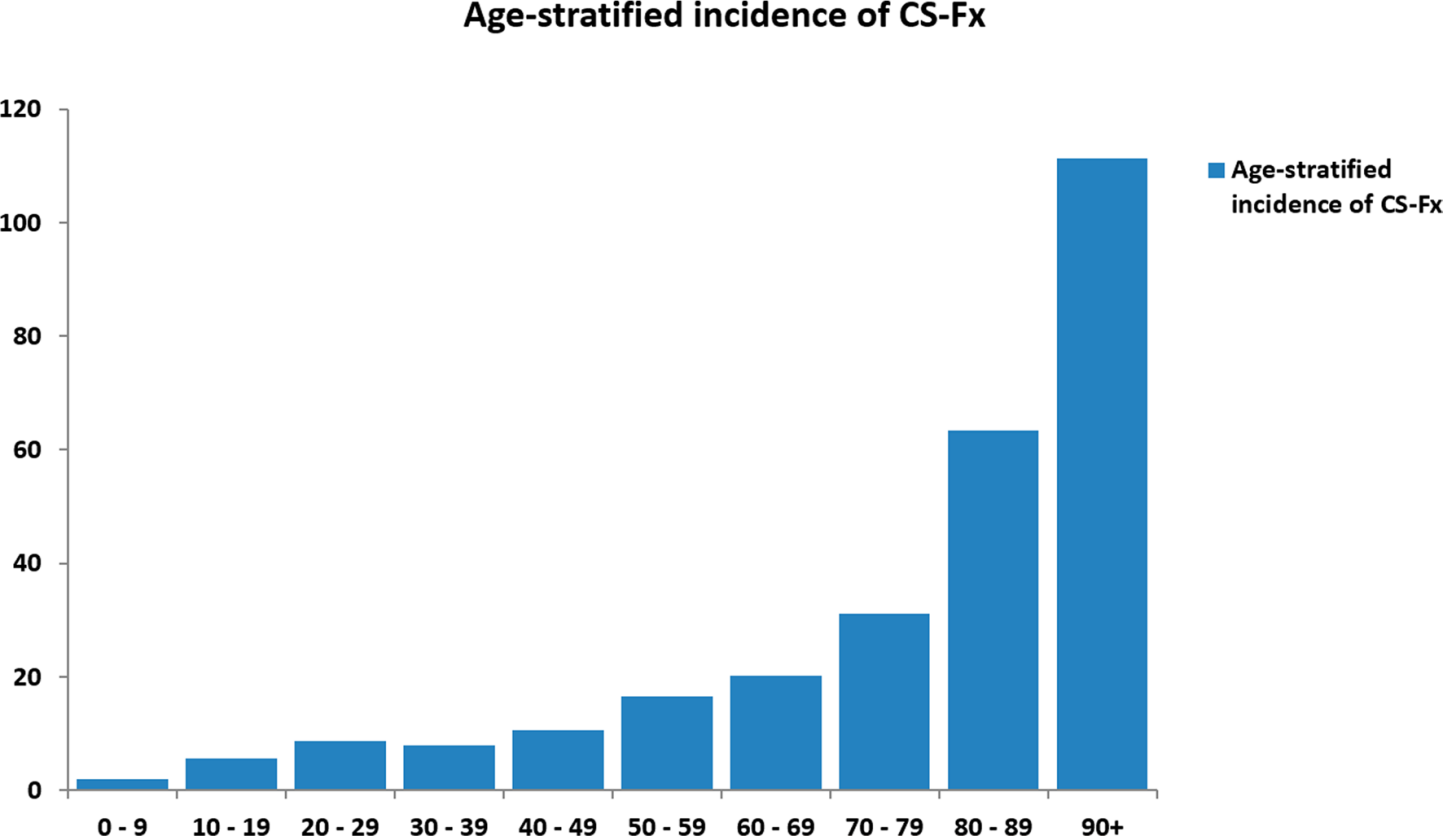
Age-stratified incidence rates of CS-Fx (count per age group/population of age group × 100,000)

**Fig. 3:**
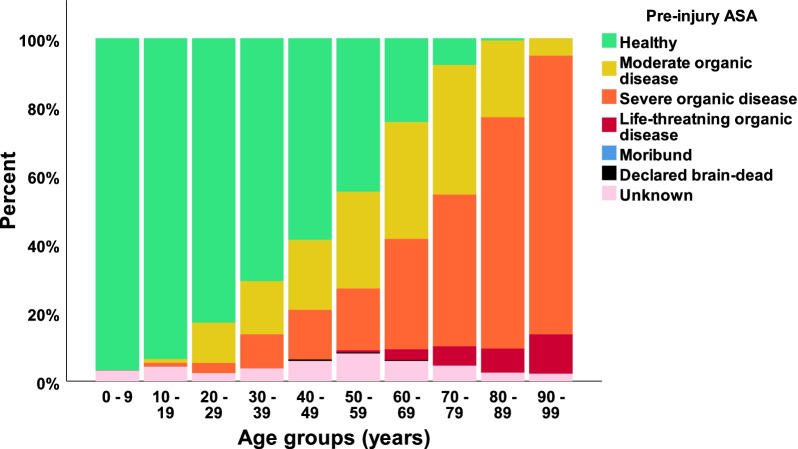
Preinjury ASA according to age group

**Fig. 4:**
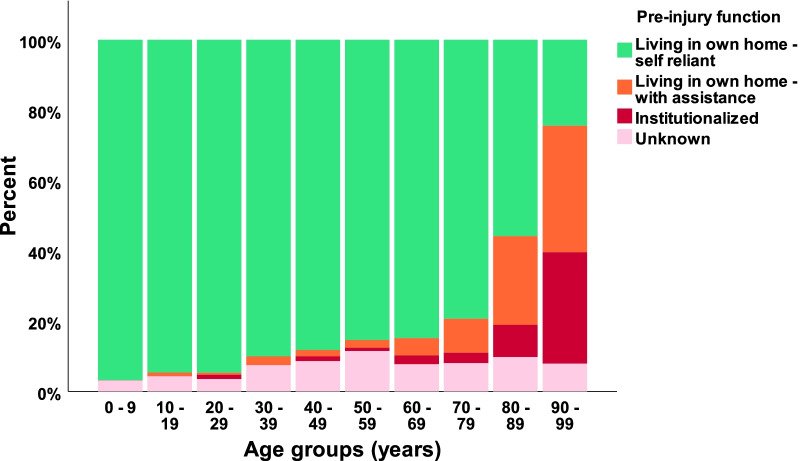
Preinjury function according to age group

In our defined population of 3.0 million people (Southeast Norway), we prospectively registered 2153 consecutive cases with CS-Fx for 5 years from 2015 to 2019. There were 2144 unique patients, of which 9/2144 (0.4%) had two separate injuries in the study period with CS-Fx. The overall crude incidence rate of CS-Fx in Norway was 14.9/100,000 person-years. Age-adjusted incidences using the standard population for Europe and the World were 15.6/100,000 person-years and 10.4/100,000 person-years, respectively. The median patient age was 62 years (range 2–100 years), 67.5% were males, and 46.3% were ≥ 65 years (WHO definition of elderly). The median age of males was significantly younger than that of females (58 years vs. 71 years) (p < 0.001) (Fig. 1). The relative incidence of CS-Fx increased significantly with age (p < 0.001) (Fig. 2). Increasing age was associated with a higher preinjury ASA score and a need for help with ADLs (p < 0.001) (Figs. 3 and 4). Of patients ≥ 65 years with a known preinjury ASA score and living status, 614/955 (64.3%) had an ASA score ≥ 3, and 265/909 (29.2%) needed assistance for ADL. Further patient characteristics are given in Table 1.

**Table 1:** Patient characteristics of all 2153 CS-Fx patients

	N (%) 2153 (100%)
Sex	
Male	1453 (67.5)
Female	700 (32.5)
Age	
0–19 years	125 (5.8)
20–49 years	552 (25.6)
50–64 years	480 (22.3)
65 + years	996 (46.3)
Comorbidity	
ASA^1^ 1–2	1269 (59.0)
ASA 3–5	783 (36.5)
Unknown	101 (4.5)
Living	
Independent living	1678 (77.9)
Dependent living	299 (13.9)
Unknown	176 (8.2)
Mechanism of injury	
Fall	1219 (56.7)
Bicycle	261 (11.9)
4 W-MVA^2^	212 (9.8)
Sport	100 (4.7)
2 W-MVA^3^	97 (4.5)
Diving	49 (2.3)
Pedestrian hit by MV	30 (1.4)
Other	185 (8.7)
Level of CS-Fx	
Only C0^4^–C2	807 (37.5)
Only C3–C7	1193 (55.4)
Both C0–C2 + C3–C7	153 (7.1)
Role OUH^5^	
Admitted OUH	1216 (56.6)
Image review and treatment advice from OUH	622 (28.7)
Follow-up OUH (primary treatment elsewhere)	315 (14.7)
Treatment	
Surgery	557 (25.9)
External immobilization alone	1398 (64.9)
None	198 (9.2)

^1^ASA: American Society of Anesthesiologists Physical Status Classification system

^2^4 W-MVA: Four-wheel motorized vehicle accidents

^3^2 W-MVA: Two-wheel motorized vehicle accidents

^4^CO: Occipital condyle

^5^OUH: Oslo University Hospital

### Trauma mechanism

The most common trauma mechanism for CS-Fx was falls (56.7%), followed by bicycle injuries (11.9%) and four-wheel motorized vehicle accidents (4 W-MVAs) (9.8%) (Table 1). The proportion of fall-related injuries increased significantly with age (p < 0.001). Of the fall-related injuries, 48.6% occurred at home, and 8.4% occurred in nursing homes or hospitals. The two dominant types of falls were the ones from the patient’s height (51.2%) and falls down the stairs (20.2%). The influence of ethanol was registered in 339/2153 (15.7%) and was seen at a stable rate in the age span from 20 to 79 years. No cases of ethanol influence at the time of injury were registered in patients < 20 years and were only rarely noted in patients ≥ 80 years. Of patients with falls from their height, 12.0% were under the influence of ethanol, while 36.6% of patients who fell down the stairs were under ethanol influence.

### Level of CS-Fx

The level of CS-Fx was only C0–C2 in 808 (37.5%), only C3–C7 in 1193 (55.4%), and both C0–C2 and C3–C7 in 153 (7.1%) (Table 1). The C0-C2 CS-Fxs were subclassified by fracture morphology or the type of dislocation in cases with dominating ligamentous injury. The subaxial CS-Fxs were described by level of “suspected instability” (Table 2). The numbers of fractures listed in Table 2 superseeded the number of cases since some cases had injuries at multiple cervical levels. The three most common injuries in the upper cervical spine were in declining order C2 odontoid Fx, C1-Fx, and occipital condyle Fx. The three most common injuries in the subaxial region resulted in “suspected instability” in declining order of levels C6/C7, C5/C6, and C4/C5. The most common fracture morphology subaxially was fracture of the facet joints, seen in 562/1346 (47%) of the subaxial fractures.

**Table 2:** Further description of C0-C2^1^ and C3-C7 (subaxial)^2^ CS-Fxs

CS-Fx main category	CS-Fx sub category	N (%)
C0-C2^1^	C0 Fx	219 (10.2)
	C1 Fx	245 (11.4)
	C2 Odontoid Fx	419 (19.5)
	C2 Hangman Fx	59 (2.7)
	C2 Other Fx	141 (6.5)
	Craniocervical dislocation	9 (0.4)
	Atlantoaxial distraction	8 (0.4)
	Atlantoaxial rotation	32 (1.5)
C3-C7 (subaxial)^2^	C2/C3 “Instability”	46 (2.1)
	C3/C4 “Instability”	175 (8.1)
	C4/C5 “Instability”	250 (11.6)
	C5/C6 “Instability”	366 (17.0)
	C6/C7 “Instability”	566 (26.3)
	C7/Th1 “Instability”	178 (8.3)

^1^C0-C2: Upper cervical region including the occipital condyle (C0), cervical vertebra C1, and cervical vertebra C2. The C0-C2 CS-Fxs are described by fracture morphology or the type of dislocation with dominating ligamentous injury

^2^C3-C7 (subaxial): Lower cervical region involving cervical vertebrae C3 to C7. The subaxial CS-Fxs are described by the level of suspected instability

### Cervical spinal cord injury (cSCI)

**Fig. 5:**
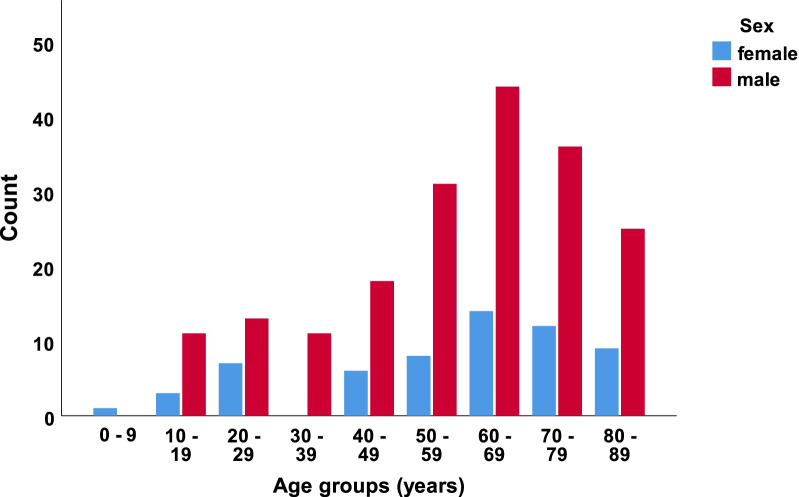
Number of patients with cervical spinal cord injury (cSCI) according to age group and sex

Concomitant cSCI was seen in 249/2153 (11.6%) of the patients with CS-Fx. AIS grade was A in 37 of the patients (14.9%), B in 35 (14.1%), C in 71 (28.5%), and D in 106 (42.5%). Thus, of the patients with cSCI, 85% had an incomplete SCI, and 15% had a complete SCI. Factors significantly associated with concomitant cSCI were male sex (p = 0.003), dependent living (p < 0.001), subaxial fractures (p < 0.001), and monotrauma (p < 0.001). Age (p = 0.4), comorbidities (p = 0.053), and injury mechanism (p = 0.1) were not associated with an increased risk of concomitant cSCI. The increased risk of cSCI in males was seen across all age groups (Fig. 5).

### Multiple trauma

Information regarding multiple traumas was missing in 133 patients. Multiple traumas were registered in 1075/2020 patients (53.2%) (Table 3). In descending order, the five most common concomitant injuries were head injury, chest injury, thoracolumbar Fx, extremity Fx, and face Fx. According to the Head Injury Severity Score (HISS), mild, moderate, and severe traumatic brain injury (TBI) was seen in 535 patients (26.0%), 118 patients (5.8%), and 79 patients (3.9%), respectively. Of the 336 patients with thoracolumbar Fxs registered, 62 required surgical fixation of the thoracolumbar Fx, and 15 had associated SCI or cauda equina injuries.

**Table 3:** Rate of multiple trauma in patients with CS-Fx. Information concerning multiple traumas was missing in 133 patients. Thus, N = 2020 patients (2153–133)

Trauma region	N (%) 2020 (100%)
Multiple trauma (any)	1075 (53.2)
Head injury^1^	721 (35.7)
Mild	525 (26.0)
Moderate	118 (5.8)
Severe	78 (3.9)
Facial Fx	204 (10.1)
Thoracolumbar Fx	336 (16.6)
Chest injury	341 (16.9)
Abdominal injury	71 (3.5)
Pelvic Fx	72 (3.6)
Extremity Fx	225 (11.1)

^1^Head injury defined as either mild, moderate, or severe according to HISS (Stein and Spettell 1995)

### Initial management

A total of 1216/2153 (56.6%) patients were admitted to OUH for initial evaluation and treatment. The remainder were managed in other hospitals within our health region after the neurosurgical team at OUH had decided that conservative treatment could be instituted at the local hospital. A few southeastern residents had initial evaluations and management performed outside our health region and came to OUH for the follow-up. The rate of admittance for initial evaluation and treatment at OUH was significantly higher for patients with concomitant cSCI than for those without concomitant cSCI (91.6% versus 51.9%) (p < 0.001) (Table 1). Of the 21 patients with cSCI not admitted to OUH for primary treatment, 15 were inhabitants of our health region who were injured outside our health region and received primary treatment outside our health region. Another five were elderly patient with mild SCI injury (AIS D) not in need of surgery and managed at local hospital after advice from us and for one old patient with AIS A we decided no treatment.

Treatment was external immobilization with a stiff collar alone in 64.9%, open surgical fixation and/or decompression in 25.9%, and no stabilization or decompression in 9.2%. Most patients in the “no stabilization or decompression group” had an isolated fracture of a spinous or transverse process. During the 5 years, a total of 557 open surgical fixations/decompressions for CS-Fx were performed in this population of 3.0 million people. The overall crude incidence rate for surgical fixation of CS-Fx in Norway was 3.7/100,000 person-years. Age-adjusted incidences for surgical fixation using the standard population for Europe and the World was 4.1/100,000 person-years and 2.8/100,000 person-years, respectively. In our series, anterior fixation alone was done in 56%, posterior fixation alone in 35%, and combined anterior/posterior fixation in 9%. The most used anterior fixation techniques was anterior cervical discectomy and fusion (ACDF) with plating, while the most used posterior fixation technique was fixation with rods and screws (Table 4). Lateral mass screws in C3–C6 were placed with freehand technique, while navigation was used for placement of lateral mass screws in C1 and pedicle screws in C2 and C6–Th1.

**Table 4:** Surgical procedures in 557 patients. The number of procedures add up to 605, since 48 patients had both anterior and posterior fixation

Surgical procedure	N
Anterior cervical discectomy and fusion (ACDF) with plating	238
Posterior cervical fixation with rods and screws (subaxial)	110
Anterior odontoid screw	63
Posterior cervical fixation with rods and screws + laminectomy (subaxial)	55
Corpectomy + autologous crista graft + anterior plating	47
Harms fixation (C1–C2 posterior cervical fixation with rods and screws)	24
Laminectomy	22
Posterior wire fixation	12
Craniocervical fixation	8
Anterior cervical discectomy and fusion (ACDF)	8
Others	18
Total	605

The rate of surgery was significantly higher for patients with concomitant cSCI than for those without concomitant cSCI (74.9% versus 19.5%) (p < 0.001) (Table 1). Management of most patients with concomitant cSCI included intensive care unit (ICU) referral to observe/support respiratory and cardiovascular function, including the aim of a mean arterial blood pressure (MAP) ≥ 85 mmHg for 5–7 days (Hadley et al. 2002; Hawryluk et al. 2015). This is reflected in the longer median hospital stay (LOS) at OUH for patients with concomitant cSCI than for those without cSCI (8 days versus 3 days) (p < 0.001).

### 90-day mortality

The overall 90-day mortality was 153/2153 (7.1%). In the univariate logistic regression analysis, the following variables were significantly associated with increased 90-day mortality: raising age, preinjury ASA score ≥ 3, dependent living, subaxial CS-Fx, concomitant cSCI, and severity of head injury (Table 5). In the multivariate logistic regression analysis, the following variables remained significantly associated with increased 90-day mortality: raising age, preinjury ASA score ≥ 3, dependent living, concomitant cSCI, and severity of head injury (Table 5).

**Table 5:** Uni- and multivariate logistic regression of variables with potential associations with increased 90-day mortality

	Summary					Univariate analysis		Multivariate analysis			
Characteristic	Nonmissing values	Alive, N = 1836^1^, N (%)	Dead < 90 days, N = 153^1^, N (%)	Dead > 90 days, N = 164^1^, N (%)	OR^2^		95% CI^2^	p value	OR^2^	95% CI^2^	p value
Age	2151	58 (40, 73)	83 (73, 90)	83 (74, 89)	1.06		1.05, 1.08	< 0.001	1.06	1.04, 1.08	< 0.001
Sex	2153										
Female		570 (31)	62 (41)	68 (41)							
Male		1266 (69)	91 (59)	96 (59)	0.69		0.49, 0.97	0.029	1.50	0.96, 2.38	0.076
Comorbidity	2052										
ASA 1–2		1222 (70)	27 (18)	20 (13)							
ASA 3–5		525 (30)	120 (82)	138 (87)	8.33		5.51, 13.0	< 0.001	3.96	2.06, 7.95	< 0.001
Living	1977										
Independent living		1534 (91)	67 (48)	77 (52)							
Dependent living		156 (9.2)	73 (52)	70 (48)	7.77		5.42, 11.1	< 0.001	2.74	1.65, 4.62	< 0.001
Level of Cs-Fx	2153										
C0-C2		642 (35)	83 (54)	82 (50)							
C0-C2 & C3-Th1		136 (7.4)	7 (4.6)	11 (6.7)	0.42		0.17, 0.86	0.030	0.62	0.19, 1.69	0.4
C3-Th1		1058 (58)	63 (41)	71 (43)	0.49		0.35, 0.68	< 0.001	0.96	0.59, 1.55	0.9
SCI—AIS grade	2152										
AIS A & B		51 (2.8)	19 (12)	2 (1.2)							
AIS C & D		158 (8.6)	4 (2.6)	15 (9.2)	0.06		0.02, 0.18	< 0.001	0.03	0.00, 0.11	< 0.001
AIS E		1627 (89)	130 (85)	146 (90)	0.20		0.12, 0.36	< 0.001	0.09	0.04, 0.21	< 0.001
Multitrauma	2020										
No		811 (47)	55 (40)	79 (55)							
Yes		927 (53)	84 (60)	64 (45)	1.37		0.97, 1.96	0.078	1.00	0.46, 2.04	> 0.9
TBI—HISS grade	2020										
Severe		49 (2.8)	28 (20)	1 (0.7)							
Mild		478 (28)	28 (20)	28 (20)	0.10		0.05, 0.18	< 0.001	0.02	0.01, 0.04	< 0.001
Moderate		96 (5.5)	14 (10)	8 (5.6)	0.24		0.11, 0.49	< 0.001	0.06	0.02, 0.16	< 0.001
None/minimal		1115 (64)	69 (50)	106 (74)	0.10		0.06, 0.17	< 0.001	0.02	0.01, 0.04	< 0.001
Thoracolumbar Fx	2020										
No		1439 (83)	118 (85)	127 (89)							
Yes		299 (17)	21 (15)	16 (11)	0.88		0.53, 1.40	0.6	1.39	0.70, 2.71	0.3

^1^Statistics presented: median (IQR); n (%)

^2^OR = Odds Ratio, CI = Confidence Interval

## Discussion

The incidence of CS-Fx in the general population of the Southeast Norway health region was 14.9/100,000 person-years, and a large fraction of the patients were elderly with significant preinjury comorbidities. The two most frequent trauma mechanisms were falls and bicycle accidents. At the time of injury, 16% of the patients were under the influence of ethanol. Treatment was external immobilization with a stiff neck collar alone in 64.9%, open surgical fixation and/or decompression in 25.9%, and no stabilization or decompression in 9.2%. Concomitant cSCI was registered in 12% and was associated with higher admittance rates to a level 1 trauma center, a higher surgical rate, and an increased LOS stay.

The overall crude incidence of traumatic CS-Fx in general Western populations is of 4–17/100,000 person-years in Canada, Finland, Germany, Ireland, Norway, the Netherlands, and Sweden (Brolin and Holst 2002; Fredo et al. 2014, 2012; Hackenberg et al. 2022; Hu et al. 1976; Kumar et al. 2018; Niemi-Nikkola et al. 1976; Roche et al. 2008). In the present study from 2015 to 2019, we estimate the overall crude incidence of CS-Fx in the general Norwegian population to be 14.9/100,000 person-years, thus confirming the results of a previous Norwegian publication covering the period of 2009–2012 (Fredo et al. 2014). For future comparison of the incidence of CS-Fx in Norway with other countries, we age-adjusted our incidence rates using the 2013 European standard population (ESP) and the 2000–2025 WHO World standard population. Age-adjusted incidences using the standard population for Europe and the World were 15.6/100,000 person-years and 10.4/100,000 person-years, respectively. Most likely, the “true” incidence of CS-Fx is slightly higher due to some undiagnosed fractures and underreporting of CS-Fx from local hospitals to the registry at the level 1 trauma hospital (OUH). We believe the three most likely reasons for undiagnosed fractures are as follows: (1) trauma victims do not contact the health care system; (2) appropriate radiological examinations are not performed; and (3) trauma victims die at the accidents’ scene. The fraction of missed cases of CS-Fx due to inappropriate radiological examinations is most likely very low at this time, as high-quality cervical CT with reconstruction has become the standard assessment for cervical injuries in all levels of health-care services in Norway. The level of underreporting of CS-Fx from local hospitals to the central registry is most likely low. The completeness of this reporting is supported by the equality of our results compared to the nationwide CS-Fx incidence in our previous publication based on data from the Norwegian Patient Registry (NPR) (Fredo et al. 2014). For the time period 2015–2019, we have not compared our CS-Fx Registry with the NPR. Such a comparison could most likely indicate the degree of underreporting of diagnosed CS-Fx from local hospitals, but cannot estimate the number of undiagnosed CS-Fx.

Most studies report a male preponderance of CS-Fx (Brolin and Holst 2002; Fredo et al. 2014, 2012; Niemi-Nikkola et al. 1976; Roche et al. 2008). This is in line with the 68% male patients in our present study. The main reason for male overrepresentation is most likely a gender difference in everyday risk-taking behavior (Pawlowski et al. 2008). The gender difference diminished with increasing age, and in the > 80-year-old age group, there were more women than men. This could be due to a combination of more osteoporosis in women and longer life expectancy for women. In a previous Norwegian publication, we found that the frequency and the relative incidence of CS-Fx were the highest among the elderly and rather rare in children (Fredo et al. 2014). The latter finding was confirmed in the present study and is similar to what has been observed in contemporary series of patients with TBI (Tverdal et al. 2020; Maas et al. 2017; Steyerberg et al. 2019).

Increasing age was associated with both more comorbidities and the need for help with ADL. Of patients ≥ 65 years of age (WHO definition of elderly), 64% had preinjury severe systemic disease, and 29% needed assistance in ADL. The increasing number of old and frail patients with CS-Fx may change the treatment strategy for some fracture types and, for many, result in prolonged acute care and increased mortality. The most common fracture in the elderly is an odontoid fracture. Surgical fixation has previously been recommended for type II odontoid fractures. We have recently documented that most of these patients can be managed with external immobilization alone, thereby avoiding a surgical procedure associated with high risk in frail older people (Rizvi et al. 2021a, 2021b, 2020).

The most common trauma mechanisms for CS-Fx were falls (57%), followed by bicycle accidents (12%) and 4 W-MVAs (10%), reflecting the rather high mean age of the patients. Of the fall injuries, 49% occurred at home, and 8% occurred in nursing homes or hospitals. The two dominating types of falls were falls from the patient’s height (51%) and falls downstairs (20%). Given the aging population worldwide, the incidence of fall-related CS-Fx is expected to continue rising unless effective fall preventive measures are taken. Defined risk factors for falls are age > 80 years, comorbidities, polypharmacy, impaired cognition, impaired hearing, and impaired vision (Lubetzky 2020; Montero-Odasso et al. 2019). To date, most studies indicate a benefit of interventions to prevent fall injuries in the elderly (Montero-Odasso et al. 2021; Bhasin et al. 2020; Liu-Ambrose et al. 2019; Tricco et al. 2017).

In Norway, bicycle injuries are a more frequent cause of CS-Fx than 4 W-MVAs. Road safety policies have had great success for reducing 4 W-MVAs, while road safety for bicyclists has lagged (Naess et al. 2020). Norwegian authorities encourage people to commute by bicycle to improve public health, decrease rush-hour traffic jams and reduce pollution. However, the increasing numbers of bicyclists, especially during rush-hour traffic, have resulted in a rising number of serious bicycle injuries (Naess et al. 2020). Bicyclists have a much higher injury risk per transported km than car occupants (Nilsson et al. 2017). Thus, road safety must be improved for bicyclists.

As for TBI patients, ethanol influence was registered in many of our patients with CS-Fx and was especially associated with falling in stairs (Tverdal et al. 2020; Steyerberg et al. 2019). Sixteen % of the CS-Fx cases were under influence of ethanol at time of injury. Ethanol is probably a risk factor for CS-Fx, but the size of the risk is difficult to estimate, since the day prevalence of ethanol intake in the Norwegian population may be as high as 12% (Norwegian Institute of Public Health https://www.fhi.no). Excessive alcohol consumption impairs cognitive, motor, and sensory functions, leading to increased injury risk. A significant focus in trauma prevention must still be increased awareness of the effects of excessive ethanol use. Hopefully, public education and increasing knowledge can reduce these alcohol-related injuries. Political legislation may also help prevent ethanol-associated injuries.

A detailed description of the CS-Fx was beyond the scope of the present study. The level of CS-Fx was C0–C2 in 38%, C3–C7 in 55%, and both C0–C2 and C3–C7 in 7%. The most frequent Fx in the upper cervical spine was C2 odontoid Fx (the most common Fx in the elderly), which is in line with other reports (Fredo et al. 2012; Rizvi et al. 2020). The most frequent C3–C7 (subaxial) Fx was a facet Fx involving level C6/C7, also in line with previous studies (Fredo et al. 2016; Sharif et al. 2020).

Concomitant cSCI was seen in 11.6% of the patients with CS-Fx, of whom 85% had incomplete cSCI and 15% had complete cSCI. Factors significantly associated with concomitant cSCI were male sex, dependent living, subaxial fracture, and monotrauma. The increased risk of cSCI observed in males across all age groups is intriguing. This can be due to a gender difference in everyday risk-taking behavior (Pawlowski et al. 2008), but may also be related to other biological sex differences like frequency of congenital spinal stenosis. Using our wide definition of multiple traumas, monotraumas were more often associated with cSCI than polytraumas. This, most likely, reflects the typical patient with a CS-Fx being an elderly person injured in a low energy fall. Due to lack of information regarding Injury Severity Score (ISS), we have not elaborated more on the association between multiple trauma and SCI. Age, comorbidities, and injury mechanism were not associated with an increased risk of concomitant cSCI. Our rate of 11.6% concomitant cSCI is substantially lower than in the past (Belirgen et al. 2013; Leucht et al. 2009; Schoenfeld et al. 2012), which is in line with more recent reports (Fredo et al. 2012; Ouden et al. 1976). This variation is mainly due to incomplete registration of “less severe” cases of CS-Fx in many studies. In addition, many reports represent subpopulations (e.g., patients admitted to trauma centers or military hospitals) and not general populations. The proportion of CS-Fx patients with concomitant cSCI may also decrease due to improvements in diagnostic neuroradiology. Today, with high-quality CT, we are diagnosing many cases of CS-Fx that were previously missed on plain X-rays. In elderly patients, the most common type of incomplete traumatic cSCI is central cord syndrome following hyperextension in patients with preinjury cervical spinal stenosis (CSS) (Epstein and Hollingsworth 2015; Nowak et al. 2009; Aarabi et al. 2013a). CS-Fx patients with cSCI are more resource-demanding for level 1 trauma centers than those without cSCI due to a higher referral rate, higher rate of open surgery, and longer LOS stay. Thus, knowledge of the expected number of these patients per year is important for hospital planning. We have a separate ongoing study to further characterize patients with traumatic cSCI concerning the level of injury, the severity of the injury, patient age and comorbidities, acute management, access to specialized rehabilitation centers, and long-term functional outcome.

Fifty-seven percent of the patients with CS-Fx in the southeastern region of Norway were admitted to OUH (level 1 trauma center) for initial evaluation and treatment. The remaining patients were managed in other hospitals within our health region when the neurosurgical team at OUH determined that conservative treatment could be instituted at the local hospital. All patients in need of cervical spine surgery were admitted to OUH, as were almost all the patients with concomitant cSCI. In total, 43% of the patients were managed at local hospitals close to their homes and without unnecessary and expensive transportation to and from the level 1 trauma center. The collaboration between the trauma center and local hospitals was efficient and smooth since the safe and fast electronic transfer of CT and MR images is available between all hospitals in our health region.

Treatment was made of external immobilization with a stiff neck collar alone in 64.9%, open surgical fixation with or without decompression in 25.9%, and no stabilization or decompression in 9.2%. The majority of patients in the “no stabilization or decompression group” had isolated fractures of a spinous or transverse process. The surgery rate was significantly higher among patients with concomitant cSCI than for those without concomitant cSCI (75% versus 20%). It is difficult to evaluate whether our rate of surgical fixation is in line with that of other countries since there are hardly any published reports of the rate of surgery for CS-Fx in a defined general population. Recent reports estimated the incidence of CS-Fx surgeries in Germany to be 3.24/100,000 person-years (Hackenberg et al. 2022) and in Finland to be 4.1/100,000 person-years (Ponkilainen et al. 1976); both were very similar to our incidence of CS-Fx surgeries of 3.7/100,000 person-years.

To perform state-of-the-art surgical fixation for CS-Fx, the surgical team must be experienced and qualified, to manage all the procedures necessary for anterior and posterior decompression/fixation (Baogui and Juwen 2019; Patel et al. 1976; Aarabi et al. 2013b). Perioperative neuronavigation is an advantage in complex cases. Given an overall crude incidence rate for surgical fixation of CS-Fx in Norway of 3.7/100,000 person-years, centralization of this kind of surgery is necessary to maintain a competent 24/7 surgical service for these patients. Emergency neurosurgery for traumatic brain injury is also centralized in Norway, and the crude incidence for such procedures in Norway is 3.9/100 000 person-years (Tverdal et al. 2022).

The overall 90-day mortality was 7.1%. In the multivariate logistic regression analysis, the following variables remained significantly associated with increased 90-day mortality: raising age, preinjury ASA score ≥ 3, dependent living, concomitant cSCI, and severity of the head injury. High mortality is closely linked to older patient age and comorbidities. As mentioned above, we have recently documented that most frail old patients with C2 odontoid Fx can be managed with external immobilization alone, thereby avoiding a surgical procedure associated with high risk in frail older people (Rizvi et al. 2021a, 2021b, 2020). However, injury preventive measures must be implemented to reduce the mortality rate significantly, e.g., fall prevention measures in the elderly.

### Strengths of the study

This is a population-based—contemporary—retrospective database study (with prospectively collected data).

### Limitations of the study

Most likely, the “true” incidence of CS-Fx in Norway is slightly higher due to some undiagnosed fractures and underreporting of CS-Fx from local hospitals to the registry, as discussed above. The external validity of this study is difficult to assess due to the lack of similar studies from other countries. The lifestyle, age distribution in the population, and environmental factors are associated with CS-Fx. Thus, the external validity of this study is limited to countries with a large proportion of elderly individuals where a fall is the dominating injury mechanism. Foreign tourists and foreign workers without a Norwegian social security number treated for a CS-Fx while in Norway are not included in this study. The lack of information on injury severity scores (ISS) in the Registry used is a limitation.

## Conclusions

This study provides an overview of the extent of the issue and patient complexity necessary for planning the health-care management and injury prevention of CS-Fx. The typical CS-Fx patient was an elderly male or female with significant comorbidities injured in a low-energy trauma. The overall crude incidences of CS-Fx and surgical fixation of CS-Fx in Southeast Norway were 14.9/100,000 person-years and 3.7/100,000 person-years, respectively.

## Data Availability

The datasets generated and/or analyzed during the current study are not publicly available due to the sensitivity of the material. The datasets can be made available from the corresponding author on reasonable request but will require permission from the Data Protection Officer at OUH.

## Abbreviations

ADL: Activities of daily living
ASA: American Society of Anesthesiologists
C0: Occipital condyle
C0-C2: Upper cervical spine
C3-C7: Subaxial cervical spine
C1-C7: Cervical vertebra 1–7
cSCI: Cervical spinal cord injury
CS-Fx: Cervical spine fracture
HSØ: The Southeastern Norway Regional Health Authority
ICU: Intensive care unit
LOS: Length of hospital stay
MAP: Mean arterial pressure
OUH: Oslo University Hospital
TBI: Traumatic brain injury
4-W MVA: Four-wheel motorized vehicle accident
